# An accurate and cost-effective alternative method for measuring cell migration with the circular wound closure assay

**DOI:** 10.1042/BSR20180698

**Published:** 2018-10-31

**Authors:** Michael L. De Ieso, Jinxin Victor Pei

**Affiliations:** Department of Physiology, Adelaide Medical School, University of Adelaide, Adelaide, SA 5005, Australia

**Keywords:** cell migration, circular wound closure, scratch assay

## Abstract

Cell migration is important in many physiological and pathological processes. Mechanisms of two-dimensional cell migration have been investigated most commonly by evaluating rates of cell migration into linearly scratched zones on the surfaces of culture plates. Here, we present a detailed description of a simple adaptation for the well-known and popular wound closure assay, using a circular wound instead of a straight line. This method demonstrates improved precision, reproducibility, and sampling objectivity for measurements of wound sizes as compared with classic scratch assays, enabling more accurate calculations of migration rate. The added benefits of the method are simplicity and low cost as compared with commercially available assays for generating circular wounds.

## Introduction

Cell migration is a multistep process that is essential for diverse life functions in multicellular and single-celled organisms, and includes both collective and individual cell movements across extracellular spaces or through tissues [1,2]. In normal physiological processes, migration enables morphogenesis, immunity, and tissue repair [2,3]; in pathological processes, migration has been linked to cancer, atherosclerosis, rheumatoid arthritis, multiple sclerosis, and others [4–9]. Understanding the mechanisms of cell migration could facilitate the development of therapeutic interventions for a wide range of diseases.

Existing literature provides a comprehensive comparison of advantages and disadvantages of approaches for measuring two-dimensional (2D) cell migration [10]. A technique commonly used for measuring 2D cell migration is the scratch wound assay. In brief, the 2D scratch wound assay involves creating a linear ‘scratch’ or wound across a confluent monolayer of cultured cells, and capturing images to measure cell migration rate by the decrease in distance across the open wound as a function of time [11,12]. Though useful, the 2D scratch wound assay has disadvantages (summarized in Table 1), stemming primarily from the fact that the scratch wound is usually longer (but not wider) than the field of view used during analysis. Without live-cell imaging facilities (to capture images in identical locations at repeated intervals), experimenters are faced with the challenge of recapturing the same position on the scratch at multiple time points without subjective error. This is especially difficult for high-throughput assays with multiwell plates, and is likely to result in reduced reproducibility of results. A second disadvantage is that typically scratch wound images are quantitated by visually estimating the positions of the boundaries of the scratch, assuming lines to approximate the walls, and measuring the distances across the gap. Manually taking multiple measurements of the gap distances at various locations is intended to reduce variability by generating an average value of the distance across the scratch [13], but the reliability is handicapped by the fact that the boundary edges are ragged; the selected positions for the boundaries will vary between samples and within samples. Analyses with the classic scratch method must be done blinded to reduce the risk of unintentional bias in the acquisition of data. Improvements on the method have used image analysis software to find lines of best fit to measure the boundaries or areas of wounds [14,15], but the scratch method is still vulnerable to variability in the image locations selected at each time point. The third consideration is that most studies with the 2D scratch wound assay have not accounted for the potentially confounding effects of cell proliferation on the apparent rate of closure of the wound, a factor that might not be addressed fully by a ‘serum-starvation’ step prior to commencing the assay [16–18].

**Table 1:** Summary of assays used previously for measuring 2D cell migration, including advantages and disadvantages

Assay	Method	Advantages	Disadvantages	Diagram
Scratch wound assay	(1) Generate confluent monolayer of cells (2) Use pipette tip to scratch a portion of cells away, leaving a ‘wound’	• Cost effective • Minimal equipment required	• Difficult to relocate exact wound sites at sequential time points without expensive live-cell imaging facilities, reducing accuracy of results	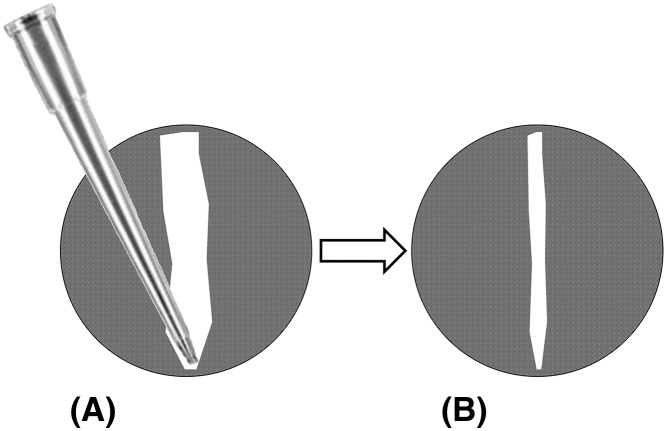
Cell exclusion zone assay with stopper	(1) Insert stopper in well prior to seeding cells (2) Allow cells to grow around the stopper (3) Remove stopper to expose circular wound	• Consistent initial wound size • High throughput • Semi-automatic	• High cost • Technically complex • Unknown effects of stopper-derived components on cell properties	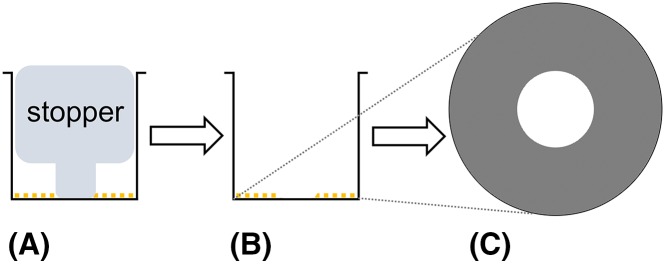
Cell exclusion zone assay with biocompatible gel	(1) Apply gel in the center of each well prior to seeding cells (2) Allow cells to grow around the gel (3) Remove gel to expose circular wound	• Consistent initial wound size	• High cost • Gel needs to be manually removed, which may alter cell or substrate properties • Low throughput (24 wells per assay)	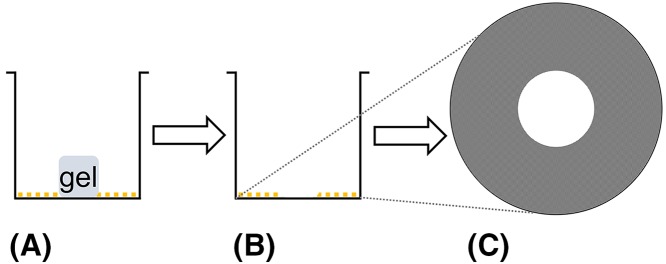

**Figure 1:**
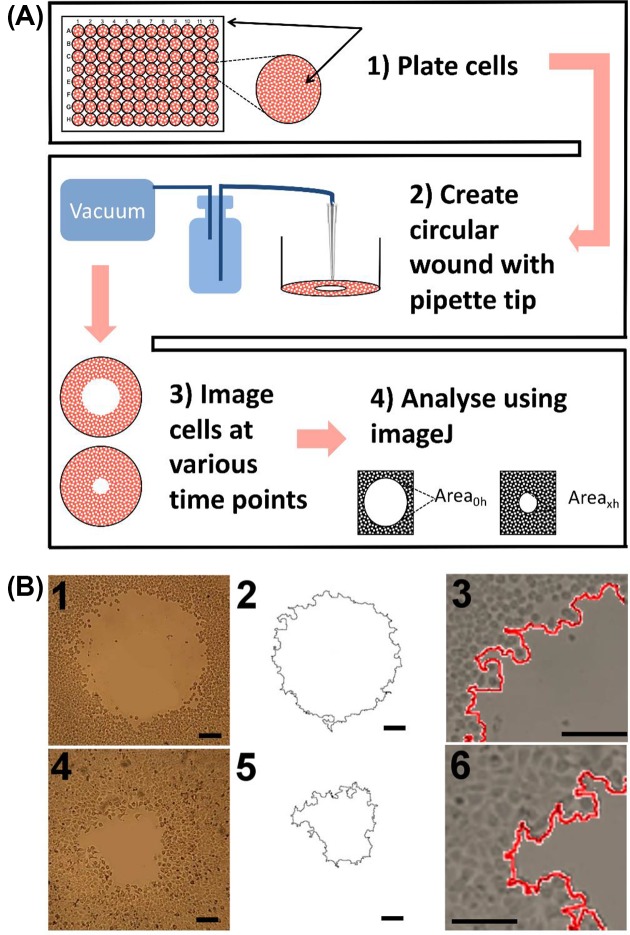
Schematic summary of procedures and examples of results for the CWCA in HT29 cells (**A**) (1) Seed the cells in a 96-well plates and grow to full confluence. (2) Connect a p10 pipette tip to a vacuum pump and gently press the end of the pipette tip perpendicularly down onto the cell monolayer (without lateral movement) to detach cells from the substratum, creating a circular wound. (3) Image the wound at various time points. (4) Measure cell migration by calculating the percent change in wound area over time, standardized to the initial area at time zero. (**B**) Raw images of the same circular wound at 0 (B1) and 24 h (B4). Outlines of circular wound perimeters at 0 (B2) and 24 (B5) h were generated by ImageJ software. Magnified superimposed views of circular wounds show outlines at 0 (B3) and 24 (B6) h, illustrating the precision of the data capture method. Black bars represent 100 µm.

**Figure 2:**
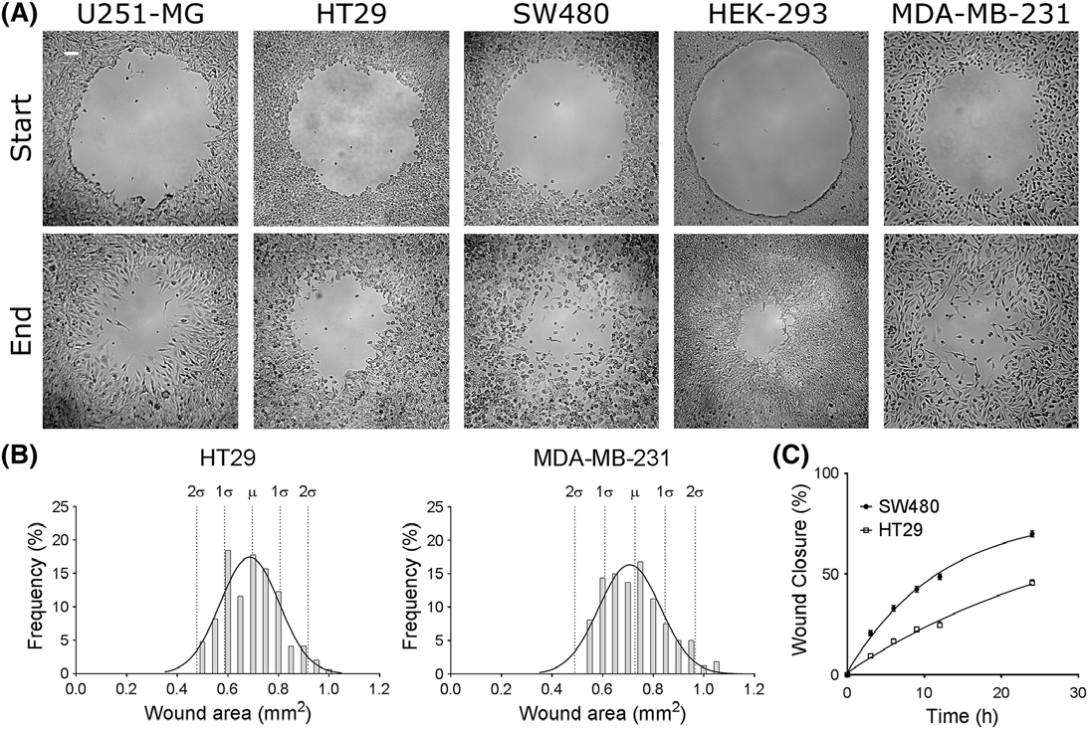
Wounds can be generated consistently for various cell lines using CWCA (**A**) Start-point represents 0 h and end-point represents various time points depending on the cell line. The end points for cell lines shown are: U251-MG 20 h, HT29 24 h, SW480 24 h, HEK-293 24 h, and MDA-MB-231 20 h. White bar represents 100 µm; the scale is consistent for all images. (**B**) Wounds were generated by two different experimenters (subjects) for two different cell lines (MDA-MB-231 and HT29), using the CWCA technique described here. The initial wound sizes were calculated (in mm^2^), and the datasets from each subject were combined for each cell line. The plots depict Gaussian distributions of the resulting initial wound areas. For -MB-231, the mean (µ) wound area is 0.728 mm^2^ (S.D. (σ) ± 0.119 mm^2^). *N*-value is 160. For HT29, µ is 0.697 mm^2^ (σ ± 0.110 mm^2^). *N*-value is 146. (**C**) Wound closure was recorded as the percent change in wound area with time (3, 6, 9, 12, and 24 h) in HT29 and SW480 cells. SW480 cells show a faster rate of migration than HT29 cells. Non-linear (sigmoidal) regression functions showed the best fit of wound closure as a function of time, yielding a correlation coefficient of *r*^2^ = 0.95 for SW480 (*n*=16), and *r*^2^ = 0.94 for HT29 (*n=*7).

The concept for generating a circular wound for measuring 2D cell migration has been previously established [19–21]. The circular wound closure assay (CWCA) permits the analyst to easily relocate the wound at any time point, and it enables accurate analysis by calculating the area or the radius of the circular wound using image analysis software. Current techniques to generate circular wounds, such as exclusion zone assays, [22] involve growing the cells around circular barriers (poly-dimethylsiloxane micropillars, stoppers, or biocompatible gels) of uniform size [21], or using a stabilized, rotating, silicone-tipped drill press to create uniform, circular wounds in an intact confluent monolayer of cells (Table 1) [19]. One advantage to these techniques is that they can generate highly consistent initial wounds; however, they are more complex and costlier than the CWCA described here. The CWCA uses a sterile 10 μl (P10) micropipette tip attached to an aspirator to remove a small circular area of cells (Figure 1). The complete wound can be reliably relocated for manual or automatic imaging at all subsequent time points. Processing images of circular wounds for analysis can be done with the freely available cross-platform Fiji (ImageJ) software [23]. Use of a mitotic inhibitor minimizes confounding effects of proliferation on apparent wound closure rates; this step is optional depending on cell type and assay duration. In summary, with this protocol easily relocatable, clean, sufficiently uniform circular wounds can be generated in diverse cell lines (Figure 2) that are amenable to streamlined computer-assisted data analysis, without costly equipment or reagents. These modifications reduce the cost and simplify the analysis of *in vitro* cell migration assays.

## Materials and methodology

### Cell lines

Lines used for the present study were: (1) human colorectal adenocarcinoma HT29 (supplied by the American Type Culture Collection (ATCC^®^), catalog number HTB-38™), and SW480 (ATCC^®^, catalog number CCL-228™); (2) human glioblastoma cell line U251-MG (supplied by the European Collection of Cell Cultures [ECACC; Salisbury, U.K.], catalog number 09063001 purchased from CellBank Australia [Westmead, NSW, Australia]); (3) mammary adenocarcinoma MDA-MB-231 (ATCC^®^, catalog number HTB-26™); and (4) human embryonic kidney HEK-293 (ATCC^®^, catalog number CRL-1573™).

### Reagents

- Cell culture medium and supplements appropriate for cell line
- 5-Fluoro-2’-deoxyuridine (FUDR) (100 ng/ml final solution)
- Lifting solution, 0.25 mM EDTA with 0.25% trypsin (2.5%, Gibco)
- Phosphate buffer saline (PBS, Gibco)

### Equipment

- Cell culture incubator at 37°C with 5% CO_2_
- Inverted light microscope with camera attachment
- Flat bottom 96-well plates
- Vacuum pump for molecular biology (Welch Laboratory, 2511B-01, 219 mmHg vacuum pressure)
- p10 pipette tips (Labcon, LC1038-290)
- Hemocytometer

### Free software

- XnConvert version 1.73 (https://www.xnview.com/en/xnconvert/#downloads)
- Fiji (ImageJ) version 1.51h (http://imagej.nih.gov/ij/)

### Procedure

Note: Perform assays under sterile conditions. See Figure 1 for short summary and example of wound and outline appearance.

**Figure 3:**
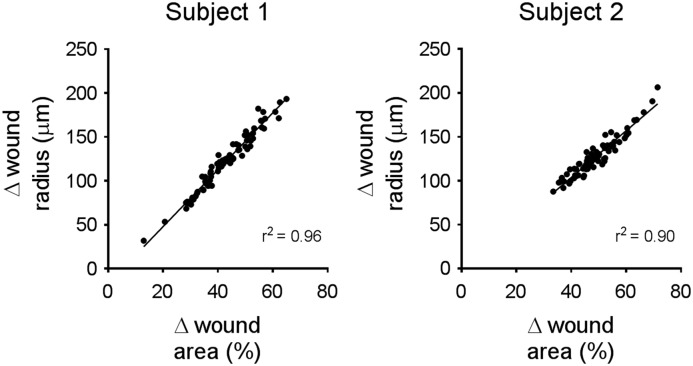
Results obtained from calculations of percent wound closure and wound radius decrease are strongly correlated Plots generated from the experimental results obtained by two different experimenters (subjects). Each experiment had various treatment groups, with some treatments exhibiting inhibitory effects on cell migration (explaining that wide range of wound closure in both plots). Analysis was done by calculating both the percentage wound closure and the change in wound radius for each wound image. The results from each method were compared and linear regression yielded a correlation coefficient of *r*^2^ = 0.96 for subject 1, and *r*^2^ = 0.9 for subject 2. *N*-value is 73 for each subject. These results suggest that both techniques of analysis produce data that is strongly correlated.

1. Passage the cells: When the cells to be used for the assay are approximately 70–80% confluent, detach the cells with trypsin and EDTA cell-lifting solution, and centrifuge cells at 125 ***g*** for 5–7 min. Resuspend cells and perform a cell count using a hemocytometer. Note: The CWCA can be used to generate wounds in diverse cell lines (Figure 2A).
2. Plate the cells: Prepare an appropriate volume of working cell solution at 5 × 10^5^ cells/ml. Plate the working cell solution at 500 μl/well for 24-well plates or 100 μl/well for 96-well plates. Incubate the plate until cells reach 90% confluency. Note: Incubation time will vary depending on the cell line.
3. Mitotic inhibitor and serum starvation: When the cells reach 90% confluency, exchange the media with FUDR-containing reduced-serum medium (1–2% serum), and incubate overnight. Use FUDR at a concentration of 100 ng/ml. Note: The use of a mitotic inhibitor is not required although recommended to reduce potential overestimation of apparent migration due to cell proliferation (Supplementary Figure S1). The serum-starvation step is essential.
4. Create wound using vacuum pump: Attach a p10 pipette tip to the end of vacuum tube (to do this, it may be necessary to first attach a p200 pipette tip to the tubing, and then overlay a p10 pipette tip on the p200 pipette tip). With medium still in the well, position the pipette tip perpendicularly above the center of the well. Gently lower the tip and make brief contact with the base to aspirate off a circular layer of cells and create a circular wound (Figure 1). Figure 2B shows the consistency of initial wound sizes generated using this technique. Note: Gentle perpendicular contact between the pipette tip and the cell monolayer is important for clean and consistent wounds. Practicing the technique in several wells prior to the first experiment is recommended (see Supplementary Figure S2 for examples of good and bad wounds). Flat pipette tips from two different vendors (Labcon, LC1038-290 and Brand Z740066) and vacuum pumps with different pressure settings (219 and 449 mmHg) have been tested in our lab with no distinctive differences in wound quality.
5. Wash the wound: Aspirate any remaining medium from the edge of the well, and wash with PBS. One wash is usually sufficient, but some cell lines will require an extra washing step to clear any residual cellular debris.
6. Apply treatments: Remove the PBS/media from wells and replace with culture medium containing the treatments or control samples that are being tested. Prepare treatments and controls in the same FUDR-containing reduced-serum medium as used previously. For example, if certain chemicals are being tested for their effect on cell migration, dissolve these chemicals at the appropriate final concentration in FUDR-containing reduced-serum medium.
7. Imaging: Using microscopy imaging facilities, capture images of each complete circular wound centered in the field of view. Once all wells have been imaged, return the plate back to the incubator until the next time point (if imaging is being performed manually). If desired, wound closure can be monitored over multiple time points (Figure 2C). The final time point for imaging depends on the cell line, as some cells migrate faster than others. Note: The maximal duration of the experiment should ensure the wounds do not fully close during the treatment period of interest.
8. XnConvert: This software can be used for batch image processing to crop to regions of interest or to change resolution of pictures.
9. Process images in ImageJ: Use NIH ImageJ software to calculate the wound area and to generate an outline of the perimeter of the wound area. The following steps illustrate how to analyze the wound area on ImageJ, and also how to use the ‘macro’ feature to semi-automate the analysis for each image, improving consistency and objectivity of measurements. The same macro settings should be used for all sampled images collected in an experiment.
  - Download and open Fiji (ImageJ).
  - Select **File>Open** and then select the image file to be analyzed.
  - Go to **Analyze>Set Scale** and input the scale information relative to your image.
  - To begin recording the macro to be used for all images, select **Plugins>Macro>Record…** A ‘Record’ box will appear, and the macro will now begin to record all following selections.
  - Select **Image>Type>8-bit.** This will convert the image to binary image.
  - Select **Process>Find Edges**.
  - Select **Process>Sharpen**.
  - Select **Image>Adjust>Threshold.** Be sure the settings are set to ‘Default’ and ‘B&W’ and untick the ‘Dark background’, and ‘Stack histogram’ boxes.
  - Move the two bars until the best clarity and contrast is achieved for the image. See image below for how the image should look following adjustment.
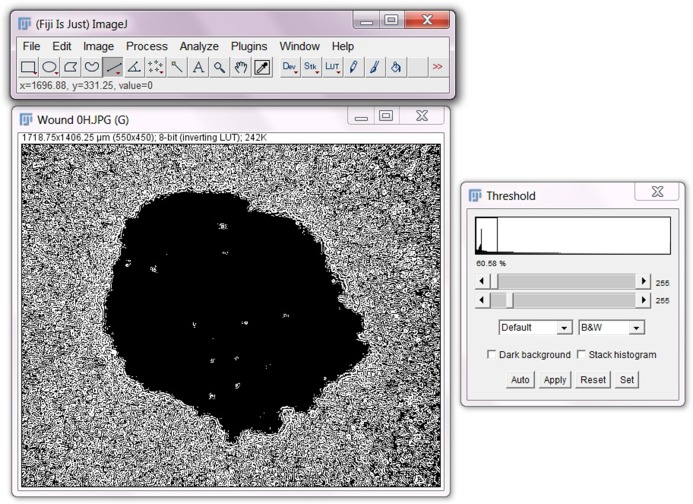
  - Select **Set** and a ‘Set Threshold Level’ box should appear. Select **OK**.
  - Now select **Apply** in ‘Threshold’ box.
  - Select **Process**>**Find Edges.**
  - Select **Image**>**Lookup Tables**>**Invert LUT**. See image below for how the image should look after LUT inversion.
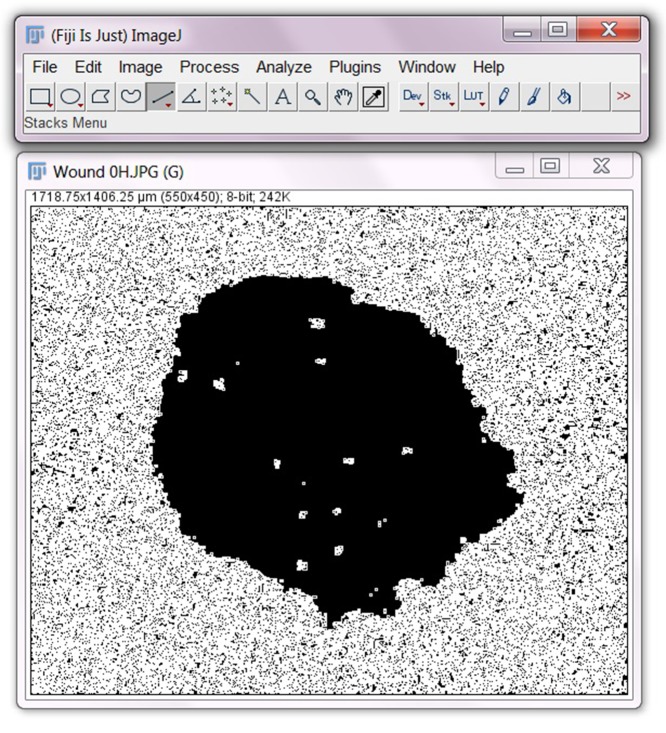
  - Select **Analyze**>**Analyze Particles…**
  - In the ‘Size (pixel∧2)’ section, select minimum and maximum pixel areas you would like the program to identify. For example, if there are artifacts (‘holes’) that are visible in the current image, and you do not want to program to mistake these ‘holes’ for wounds, it is important to input the range of areas within which wounds are likely to fall. Try ‘2000-Infinity’ to begin, and adjust accordingly. If the program is detecting ‘holes’ that are not wounds, increase the first value. If the program is not detecting anything at all, including wounds, decrease the first value.
  - Set ‘Circularity’ to ‘0.00–1.00’.
  - In the ‘Show’ section, choose ‘Bare Outlines’ to generate an outline of the wound.
  - Be sure ‘Summarize’ is ticked to generate data of the wound area.
  - Select **OK**.
  - A summary box will appear, which will include the area value of the wound to be used for further statistical analysis. An outline of the wound will also appear. See below image for summary and outline following this step.
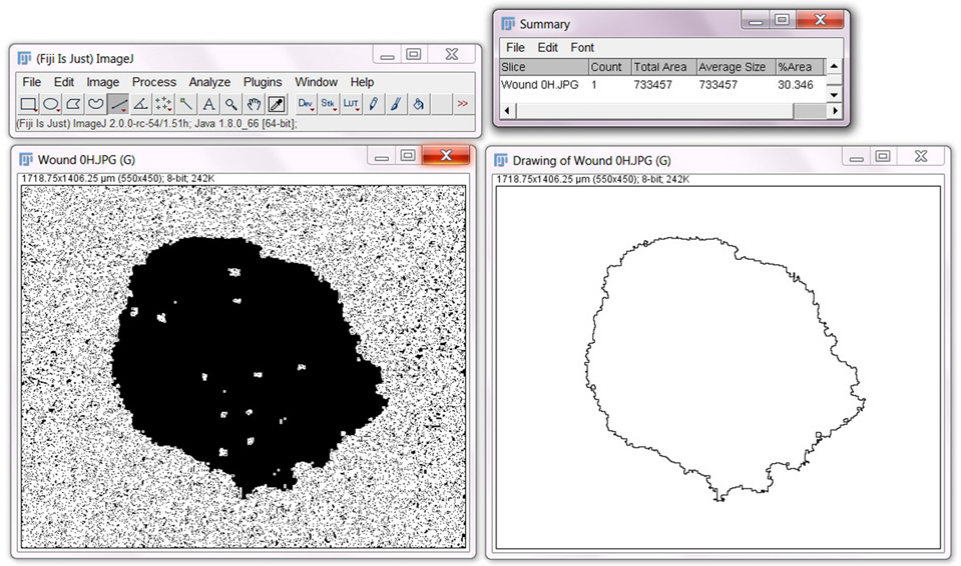
  - Find the ‘Record’ box for the macro and select** Create**.
  - A new box will appear labeled, ‘Macro.ijm’. In this box, select **Save As**, and save as a .txt file.
  - Now go to **Plugins**>**Macros**>**Install…**
  - Find and select the .txt macro file from step (v).
  - Open a new wound image for analysis.
  - Select **Plugins**>**Macros**>‘**Your Macro**’. Your macro should be located at the bottom of the dropdown box.
10. Check for initial wound size consistency: Run an ANOVA statistical test to confirm the absence of significant differences between the initial wound areas across all the control and treatment groups in an experiment. This rules out the possibility that differences in wound closure observed between treatment groups were an indirect result of initial wound size.
11. Analyze wound closure: The wound area measured at time zero (the start of the treatment) serves as a reference point for standardization. Subsequent samples can be evaluated in different ways to estimate the magnitude of cell migration. One method is to calculate the radius of the initial wound minus the radius of the end wound. This method determines distance moved but assumes circularity of the wound shapes. A second method is to calculate the final wound area as a percentage of the initial wound area. This method requires consistency of initial wound sizes, but is more tolerant of non-circular wounds. The percent closure method has been the analysis of choice for published work [24,25]; however, results from both methods show a robust correlation, demonstrating reliability (Figure 3).

## Conclusion

This CWCA technique provides a simple and reliable alternative method with distinct advantages over older methods such as the scratch wound assay or cell exclusion zone assays. Accurate data measurements enable straightforward objective computer-assisted analyses. This simple adaptation of a well-established protocol generates results that are comparable in consistency and quality to expensive commercial options and supports relatively high-throughput screening of novel therapeutic agents that regulate cell migration rates [24,25]. The main limitation of CWCA is that manual wound generation can yield higher variability in initial wound sizes and shapes as compared with cell exclusion zone or silicone-tipped drill-wounding methods; however, this limitation exists for any assays involving the manual generation of wounds. Variability is reduced with practice. Ruling out the potential impact of variability is addressed by running an ANOVA statistical test on initial wound sizes across all treatment groups in a given experiment. Absence of a significant difference rules out non-specific effects of initial wound sizes on measures of closure. Analyzing data by determining wound radius change, as opposed to percentage wound closure, is less sensitive to initial wound size, but more sensitive to the circularity of wound shape; however, both methods are reliable. In summary, this protocol offers a quality advance in methodology that is possible without specialized equipment or costly resources. Cutting edge research on cell migration can be carried out by laboratories, including those located in developing countries where research funding and facilities might be limited.

## Supporting information

**Supplementary Figure 1:** Analyses of proliferation with and without mitotic inhibitor FUDR. For the proliferation assay, cells were plated at 10^5^ cells/ml in a flat-bottom 96-well plate in 2% serum DMEM culture medium with and without 100ng/mL FUDR for 24 hours. Four images were acquired for each treatment (one image per well), standardized with XnConvert software, and used to count the total numbers of individual cells in each field of view. FUDR (100ng/mL) significantly reduced cell proliferation measured at 24 hours in HT29 and SW480 colorectal adenocarcinoma cells (p<0.0001, n=4).

**Supplementary Figure 2:** Examples of high and low-quality wounds. Images of cultured HT29 cells in two wells of a 96-well plate, immediately after wounding. (A) A high-quality wound, with a clean wound area and well-defined border, in a uniform background of cells grown to near 100% confluence. (B) A low-quality wound (inadequate for further analysis) littered with cellular debris, ragged borders, and gaps in the cell monolayer, due to lack of confluency as well as inadequate contact with the suction p10 tip onto the well floor.

## Abbreviations

CWCA: circular wound closure assay
FUDR: 5-Fluoro-2’-deoxyuridine
2D: two-dimensional
