# Supplementary material for: An accurate and cost-effective alternative method for measuring cell migration with the circular wound closure assay
Source: Biosci Rep. 2018 Oct 31;38(5):BSR20180698. doi: 10.1042/BSR20180698 (PMC6209583; doi:10.1042/BSR20180698)
Supplement: Supplementary file 1 [file bsr20180698_Supp1.pdf]

## 10 Supplementary Figures

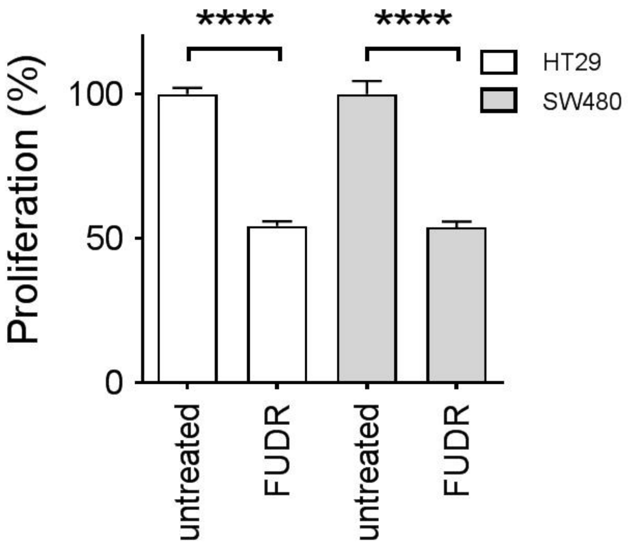


**Supplementary Figure 1: Analyses of proliferation with and without mitotic inhibitor FUDR.** For the proliferation assay, cells were plated at $10^5$ cells/ml in a flat-bottom 96-well plate in 2% serum DMEM culture medium with and without 100ng/mL FUDR for 24 hours. Four images were acquired for each treatment (one image per well), standardized with XnConvert software, and used to count the total numbers of individual cells in each field of view. FUDR (100ng/mL) significantly reduced cell proliferation measured at 24 hours in HT29 and SW480 colorectal adenocarcinoma cells ($p<0.0001$, n=4).

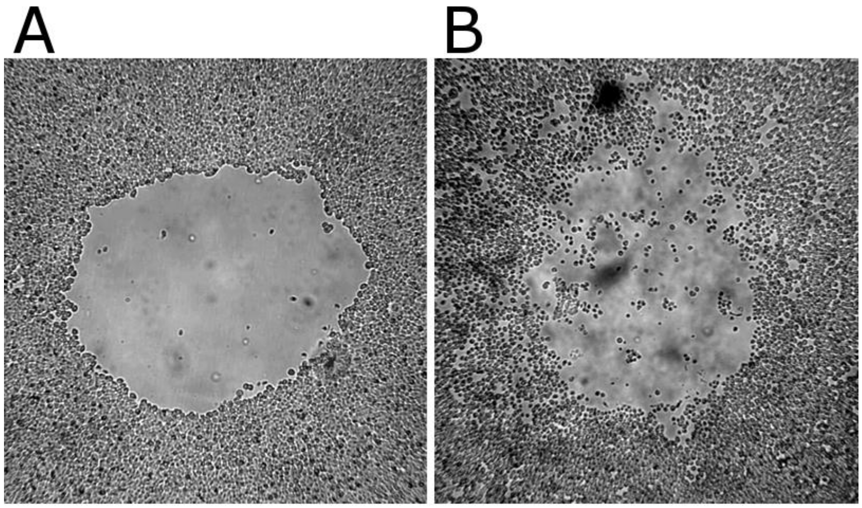


**Supplementary Figure 2: Examples of high and low-quality wounds.** Images of cultured HT29 cells in two wells of a 96-well plate, immediately after wounding. (A) A high-quality wound, with a clean wound area and well-defined border, in a uniform background of cells grown to near 100% confluence. (B) A low-quality wound (inadequate for further analysis) littered with cellular debris, ragged borders, and gaps in the cell monolayer, due to lack of confluency as well as inadequate contact with the suction p10 tip onto the well floor.
